# Highly sensitive broadband photodetector based on PtSe_2_ photothermal effect and fiber harmonic Vernier effect

**DOI:** 10.1515/nanoph-2025-0291

**Published:** 2025-10-29

**Authors:** Yinghui Gao, Yinping Miao, Xiaolan Li, Wangyang Nie, Yanxi Wang, Zhuoyang Han, Youlian Wang, Xuqi Wang, Rui Yang, Ran Wang, Jianquan Yao

**Affiliations:** Tianjin Key Laboratory of Film Electronic and Communicate Devices, School of Integrated Circuit Science and Engineering, Tianjin University of Technology, Tianjin 300384, China; Key Laboratory of Quantum Optics and Intelligent Photonics, School of Science, Tianjin University of Technology, Tianjin 300384, China; School of Integrated Circuit Science and Engineering, Tianjin University of Technology, Tianjin 300384, China; Microelectronics Instruments and Equipment R&D Center, Institute of Microelectronics of the Chinese Academy of Sciences, 3 Beitucheng West Road, Beijing, 100029, China; School of Integrated Circuits, University of Chinese Academy of Sciences, No. 19(A) Yuquan Road, Beijing, 100049, China; College of Precision Instrument and Opto-Electronics Engineering, Institute of Laser and Opto-Electronics, Tianjin University, Tianjin 300072, China

**Keywords:** platinum diselenide (PtSe_2_), Fabry–Pérot interferometer (FPI), photodetector, photothermal effect, polydimethylsiloxane (PDMS), Vernier effect

## Abstract

Platinum diselenide (PtSe_2_), a two-dimensional material, has shown exceptional promise in photodetection applications because of its air stability, high carrier mobility, and layer-tunable bandgap. However, conventional photoconductive modes face challenges with high dark currents. To address this limitation, all-optical fiber detection technology with high sensitivity and response, has emerged as a promising approach for developing PtSe_2_, based devices. In this study, a high-sensitivity broadband photodetector based on PtSe_2_ with a cascaded Fabry–Pérot interferometer (FPI) is proposed, which utilises the broad spectral absorption property of PtSe_2_ and the interference enhancement mechanism of cascaded F–P cavities to achieve high-sensitivity broadband photodetection. The experimental results show that the detector has excellent spectral response in the 808–1,550 nm band, with a sensitivity of 3.867 nm/mW at 980 nm and a response time of 37.43 ms/129.17 ms. The sensitivity at 1,550 nm is up to 134.014 nm/mW, with a response time of 75.74 ms/28.66 ms. The double matching of 0.8 eV energy and PtSe_2_ material, which is situated in the range of the material intrinsic absorption peak (1,200–1,600 nm), is responsible for the excellent sensitivity at 1,550 nm. It is also highly matched with the interband jump energy level, which generates more hot carriers per unit optical power and thereby increases the photothermal conversion efficiency. This study provides a new solution for the design of high-sensitivity, ultra-wideband optical fiber photodetectors, which has important potential applications in optical communications, environmental monitoring, and sensing.

## 1 Introduction

The emerging group 10 transition metal diselenide (TMD) two-dimensional material, platinum diselenide (PtSe_2_), has attracted much attention in the semiconductor field in recent years due to its unique electronic and optical properties [1], [2], [3]. Compared with conventional 2D materials such as graphene [4] and TMDs [5], PtSe_2_ exhibits excellent electrical properties, including high carrier mobility, tunable bandgap, and good air stability [6]. In addition, the bandgap of PtSe_2_ can be tuned by the number of layers, from semiconducting properties in a single layer to semi-metallic properties when multilayered, which provides great flexibility for its application in optoelectronics. Due to its unique electronic structure and tunable optical properties, PtSe_2_ shows great potential in photodetectors, field effect transistors (FETs), and solar cells [7], [8].

In particular, PtSe_2_ has attracted much attention in the field of photodetectors due to its unique wide spectral response range and high responsivity. In 2023, Kim et al. fabricated PtSe_2_-based photodetectors on boron hydride using molecular-beam epitaxy, with sensitivity up to 13.2 mA/W under 405 nm UV illumination [9]. In 2024, Ji et al. proposed a PtSe_2_-based photodetector based on a uni-oriented PtSe_2_ PtSe_2_/Si 2D-3D pin photodetector with light n-doped Si intercalation, with a response speed of 2.2/11.8 μs under 532 nm illumination and a sensitivity of 37 mA/W under 980 nm illumination. In recent years, PtSe_2_ photodetectors with monolayers [10], Si heterojunctions [11], and arrayed heterojunctions [12] have successfully demonstrated their unique performance advantages. However, PtSe_2_ photodetectors with conventional photoconductive mode of operation are limited by high dark current, relatively low responsivity, and slow response [13]. In contrast, fiber-based all-optical detectors not only retain the intrinsic advantages of fiber-optic systems, including high response speed [14], [15] and exceptional sensitivity [16], but also demonstrate a unique “long-range effect” [17]. This phenomenon significantly enhances the light absorption of the material, which provides an important technological insight for the development of all-optical detectors based on PtSe_2_. This makes PtSe_2_-based all-optical photodetectors an urgent research direction in the current field.

It is worth noting that the structural design of optical fiber devices has a decisive impact on their performance. Interferometric fiber optic devices show significant advantages in precision photonic sensing and optical communication systems due to their unique light wave interference principle, high sensitivity, resistance to electromagnetic interference, and adaptability to complex environments [18]. Typical structures include Fabry–Pérot interferometer (FPI), Michelson interferometer, Mach–Zehnder interferometer (MZI), Sagnac interferometer, etc. Among them, fiber Fabry–Perot cavities (fiber F–P cavities) consist of two highly parallel reflective end surfaces, which can enhance the optical signals through multiple reflections and form interference spectra. Their simple structure and neat spectra enable precise control of optical range differences [19] and show stable operating performance in various complex environments [20]. These combined advantages have made F–P cavities an indispensable and important part of many research fields, and they have been widely used in the measurement of various physical quantities such as pressure [21], curvature [22], and humidity [23]. However, traditional photodetectors with a single F–P cavity structure are limited by mode noise and resonance peak broadening, which are the key bottlenecks for their performance improvement. Thus, the sensitivity needs to be improved.

The Vernier effect breaks through the physical limit of the traditional single-cavity through the optical amplification mechanism, which provides a new idea for high-sensitivity photodetectors [24], [25], [26]. The physical nature of the Vernier effect is the optical beat-frequency effect triggered by the free spectral range (FSR) difference between two interfering cavities, and the small optical range change Δ*L* can be amplified into a significant wavelength shift. Recent advancements in multi-cavity Fabry–Pérot (F–P) sensor design, leveraging the traditional Vernier effect (TVE), have demonstrated remarkable progress in overcoming fundamental performance limitations of conventional sensors. By effectively implementing optical field superposition and interference mode coupling mechanisms, these systems achieve enhanced sensing capabilities through Vernier magnification effects, enabling the development of highly sensitive and compact photonic sensors [27], [28], [29]. Particularly, in the process of breaking through the performance boundary of traditional Vernier effect, harmonic Vernier effect (HVE) has been introduced into the sensing field as an innovative mechanism, which can achieve an order of magnitude increase in sensitivity based on the existing one [30]. Therefore, cascaded F–P cavities based on the harmonic Vernier effect are suitable as the infrastructure of photodetectors to support their needs for efficient and accurate detection in variable environments.

This work proposes a photodetector based on PtSe_2_ and the harmonic Vernier effect. The detector adopts the SMF-PtSe_2_-air-SMF(SMF: single mode fiber) structure, where FPI1 is a sensing cavity formed by PtSe_2_ mixed with PDMS, and FPI2 is an air-filled reference cavity. The harmonic Vernier effect is obtained by changing the air cavity length of the cascade optical fiber FPI, so that the optical range reaches several times (>1) of the sensing cavity. PtSe_2_ is heated by the external light, which leads to the thermal expansion of the PDMS, so that the length of the sensing cavity becomes longer. The length of the reference cavity becomes shorter. The inclination of their spectral interferences is shifted to the opposite direction, respectively, achieving an enhanced harmonic Vernier effect and multiplying the sensitivity. The device combines the photosensitive property of PtSe_2_ and the optical interference enhancement mechanism of cascaded F–P cavities. It provides a revolutionary solution for high-precision optical detection with its simple structure, high sensitivity, and fast response time.

## 2 Sensor fabrication and principle

**Figure 1:**
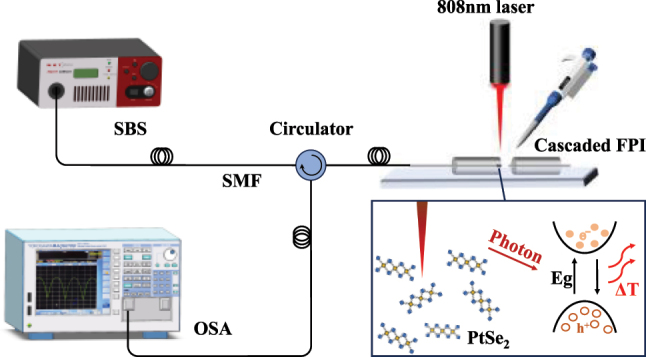
Light detection schematic and structure of the optic fiber integrated photodetector.

The structure of the experimental setup is shown in Figure 1. The supercontinuum broadband source (SBS; NKT Photonics, 600–1,700 nm) emits broad-spectrum light in the wavelength range of 600 nm–1,700 nm, and the beam is transmitted through a single-mode optical fiber and then enters into the cascade F–P cavity via a circulator. After multiple reflections of the incident light in the F–P cavity, the optical signal carrying the interference information is returned through the annulus, and the interference spectrum is finally displayed in a spectrometer optical spectrum analyzer (OSA; AQ6317D, YOKOGAWA). All measurements were conducted in a dark enclosure, and the ambient temperature was maintained at 25 ± 1 °C using laboratory air conditioning to minimize the effects of stray light and thermal fluctuations on the experimental results.

The physical structure of the cascade F–P cavity is encapsulated and supported by a quartz capillary tube. Specifically, the cascade F–P cavity consists of two parts: the left side is the sensing cavity, composed of PtSe_2_ mixed with Polydimethylsiloxane (PDMS) material (see Supplementary Material, Figure S1), and the right side is the reference cavity, composed of air medium. The preparation process is as follows: firstly, 2 mg of PtSe_2_ was uniformly dispersed in 0.1 mL of PDMS colloid to form a composite colloid with a concentration of 0.2 mg/mL, which was selected as an optimal balance between transparency and dispersion stability (see Supplementary Material, Figure S2). The prepared composite colloid was then deposited near the end face of the left SMF using a pipette gun, and the position of the optical fiber in the capillary was fine-tuned while monitoring the characteristic peak evolution of the reflectance spectra and controlling the sensing cavity length of *L*. During the construction of the reference cavity, the second section of the SMF was moved to form an air-mediated cavity at the right side of the sensing cavity. The right SMF was controlled to adjust the reference cavity length so that its optical range was twice that of the sensing cavity. This optical range matching design significantly improves the sensing sensitivity by stimulating the higher-order harmonic Vernier effect through the 2:1 FSR ratio relationship.

**Figure 2:**
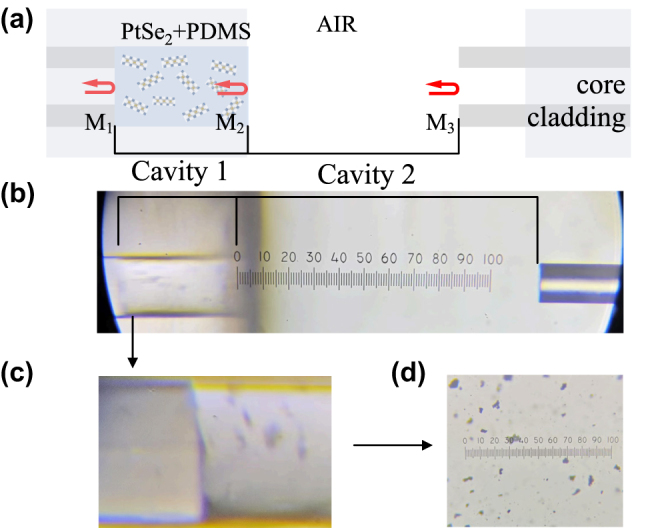
Cascade FPI structure diagram. (a) Schematic diagram of the cascade FPI structure; (b) physical diagram of the cascade FPI structure; (c) enlarged view of the physical cold splice; (d) diagram of the PtSe_2_ addition concentration.

As shown in Figure 2(a), the photodetector is based on a cascaded F–P cavity with PtSe_2_ as the photo-detection material. The sensing cavity consists of PtSe_2_ mixed with PDMS, and the detector is fabricated by cold splicing polymer gel PDMS with an optical fiber [31]. PDMS is a silicone-based elastomer with high transparency, good thermal conductivity, and chemical stability, and it is suitable for combining with other materials [32]. In this paper, it will be combined with PtSe_2_ to form a photosensitive part as a medium for the sensing cavity. Figure 2(b) shows the structure under the microscope. Since FSR = *λ* ^2^/2*nL*, the experimentally measured cavity lengths of the sensing cavity (Cavity 1) and the reference cavity (Cavity 2) are *L*_1_ = 40 μm and *L*_2_ = 115 μm, respectively, and combining with *n*_1_ = 1.4070 and *n*_2_ = 1 can be calculated as the optical range length, OPL_1_. OPL_2_ is 56.28 nm and 115 nm, respectively, and OPL_2_ = 2.04·OPL_1_; therefore, the harmonic Vernier effect can be obtained. The local magnification of SMF cold-spliced PtSe_2_ is shown in Figure 2(c), and its added concentration is shown in Figure 2(d).

**Figure 3:**
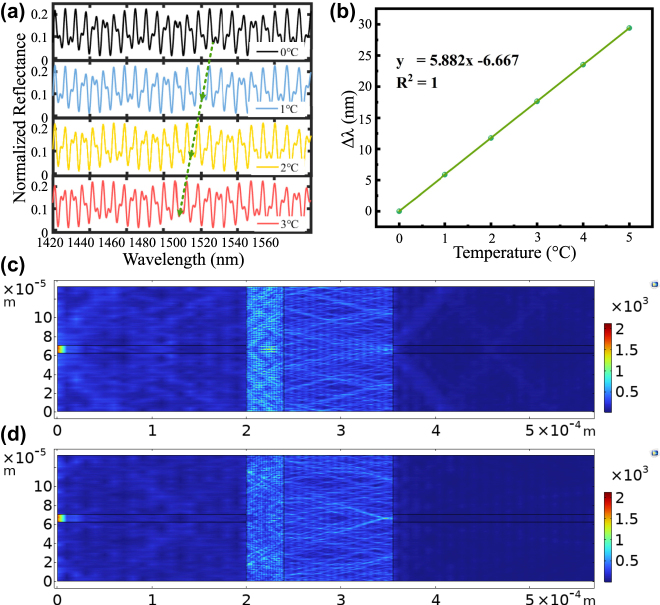
Device simulation results are plotted. (a) Simulated reflectance spectra at different temperatures (0 °C, 1 °C, 2 °C, 3 °C); (b) linear fit of wavelength drift; (c) surface: electric field mode, first wave (V/m); (d) surface: electric field mode, second wave (V/m).

When incident light propagates from the SMF core into PDMS, the refractive index (RI) contrast between the SMF core and PDMS induces Fresnel reflection at interface M_1_, generating a reflected beam (*I*_1_). The transmitted residual light then reaches the PDMS-air interface (M_2_), where the RI difference between PDMS and air causes a second Fresnel reflection, producing another reflected beam (*I*_2_). At this point, the residual light will propagate through the air until the reflecting surface M_3_, at which time the air and the SMF fiber core have different RIs and reflect the reflected beam with intensity *I*_3_. The three beams of light interfere and pass through the circulator to obtain an interference spectrogram at the spectrometer. Figure 3(a) shows the simulation of the interference spectrum. It can be seen that when the temperature changes from 0 °C to 3 °C, the interference spectrum has a very obvious blue shift due to the thermal expansion effect (TEE) of PDMS. The linear fitting plot of its wavelength drift is shown in Figure 3(b). Figure 3(c) shows the first wave of the electric field mode on the surface of the device structure, that is, the fundamental mode of the surface electromagnetic wave, whose electric field is distributed along the surface of the device, and the energy is mainly concentrated near the interface and exponentially decays into the right F–P cavity. Figure 3(d) shows the second wave of the surface electric field mode of the device structure, that is, the first-order mode of the surface electromagnetic wave, and compared with Figure 3(c), the loss of the second wave is larger. From Figure 3(c) and (d), it can be seen that the light emitted from the light source can be effectively reflected through the proposed device, which proves that the device is feasible.

A laser beam is placed above the cascade FPI structure as a pump light, and when the laser light starts to be irradiated, the photon energy is absorbed by PtSe_2_, and heat is released due to photothermal conversion. Due to the large thermal expansion effect of the PDMS, the temperature change will lead to the deformation of the PDMS, which in turn will make the cavity length of the sensing cavity longer, the reference cavity length shorter, and the spectral interference inclination angle shifted in the opposite direction, respectively.

We can roughly calculate the reflection coefficients *R*_1_, *R*_2_, and *R*_3_ for the three reflecting surfaces M_1_, M_2_, and M_3_ as [33]:

(1) $$R_{1}={\left(\frac{n_{s}-n_{1}}{n_{s}+n_{1}}\right)}^{2},\;R_{2}={\left(\frac{n_{1}-n_{2}}{n_{1}+n_{2}}\right)}^{2},\;R_{3}={\left(\frac{n_{2}-n_{s}}{n_{2}+n_{s}}\right)}^{2}$$

where *n*_*s*_ is the refractive index of the SMF fiber core material, *n*_1_ is the RI of the PtSe_2_ mixed PDMS in the sensing cavity, and *n*_2_ is the RI of the air. The light received from the circulator is partially reflected by M_1−3_ as it propagates through the entire detector structure, and the reflectance spectral function of the three reflecting surfaces can be expressed as [34]:

(2) $$E_{1}=\sqrt{R_{1}}E_{0}$$

(3) $$E_{2}=\sqrt{R_{2}}\left(1-R_{1}\right)\left(1-\alpha_{1}\right)E_{0}\exp\left(-2j\varphi_{1}\right)$$

(4) $$\begin{array}{ll} E_{3} & =\sqrt{R_{3}}\left(1-R_{1}\right)\left(1-R_{2}\right)\left(1-\alpha_{1}\right)\left(1-\alpha_{2}\right)E_{0}\\ & \;\times \exp\left[-2j\left(\varphi_{1}+\varphi_{2}\right)\right] \end{array}$$

where *E*_0_ is the incident electric field component. *φ*_1_, *φ*_2_ denote the optical range difference, *φ*_1_ is (2*πn*_1_ *L*_1_/*λ*), *φ*_2_ is (2*πn*_2_ *L*_2_/*λ*). *L*_1_, *L*_2_ denote the cavity lengths of the sensing cavity and the reference cavity, respectively, and *λ* is the wavelength of the light in the free space. *α*_1_, *α*_2_ denote the transmission loss factors of M_1_, M_2_, respectively. The total electric field of the reflected light can be considered as the sum of the electric fields reflected by these three mirrors, and therefore, the total reflected light intensity *I*_1_ of the detector is denoted as:

(5) $$I_{1}={\left|\frac{\left(E_{1}+E_{2}+E_{3}\right)}{E_{0}}\right|}^{2}$$

The free spectral range (FSR) represents the distance between two neighbouring peaks, and the FSR of FPI_1_ and FPI_2_ can be expressed as:

(6) $$\text{FS}{\text{R}}_{1}=\frac{\lambda_{1}^{2}}{2n_{1}L_{1}}$$

(7) $$\text{FS}{\text{R}}_{2}=\frac{\lambda_{2}^{2}}{2n_{2}L_{2}}$$

where *λ*_1_ and *λ*_2_ are the wavelengths of two neighbouring maxima (or minima). When the optical range lengths (OPLs) of FPI_1_ and FPI_2_ are close but not equal, they will be generated in the spectrum by the superposition of the sensing interferometer spectra and the reference interferometer spectra, that is, the TVEs. The FSR of the periodic envelope is expressed as follows [35]:

(8) $$\begin{array}{ll} \text{FS}{\text{R}}_{\text{envelope}} & =\left|\frac{\text{FS}{\text{R}}_{1}\times \text{FS}{\text{R}}_{2}}{\text{FS}{\text{R}}_{1}-\text{FS}{\text{R}}_{2}}\right|=\left|\frac{\lambda_{1}\lambda_{2}}{2\left(n_{1}L_{1}-n_{2}L_{2}\right)}\right|\\ & =\left|\frac{\lambda_{1}\lambda_{2}}{2\left(\text{OP}{\text{L}}_{1}-\text{OP}{\text{L}}_{2}\right)}\right| \end{array}$$

An HVE occurs when OPL_2_ (the OPL of FPI_2_) and OPL_1_ (the OPL of FPI_1_) are approximately multiplicative (*j* + 1 times). The OPL_2_ can be expressed as:

(9) $$\begin{array}{ll} \text{OP}{\text{L}}_{2} & =\left(j+1\right)\text{OP}{\text{L}}_{1}+\left(\text{OP}{\text{L}}_{1}-\text{OP}{\text{L}}_{2}\right)\\ & =\left(\frac{j}{2}+1\right)\text{OP}{\text{L}}_{1},\;j=0,1,2,\ldots \end{array}$$

An important feature of the Vernier effect is the magnification factor (*M*), that is, the sensitivity of the structure is magnified by a factor of *M* compared to an FPI with a single cavity, defined as:

(10) $$M=\frac{\text{FS}{\text{R}}_{\text{envelope}}}{\text{FS}{\text{R}}_{1}}=\left|\frac{n_{1}L_{1}}{n_{1}L_{1}-n_{2}L_{2}}\right|=\left|\frac{\text{OP}{\text{L}}_{1}}{\text{OP}{\text{L}}_{1}-\text{OP}{\text{L}}_{2}}\right|$$

With the advancement of nanotechnology, photothermal nanomaterials have acquired enhanced light-harvesting and photothermal conversion capabilities [36]. As a group-10 TMD, PtSe_2_ exhibits exceptional thermo-optical effects [37], enabling broadband photon absorption from deep ultraviolet to mid-infrared wavelengths. This wide spectral responsivity allows efficient conversion of captured light energy into localized thermal gradients through photothermal processes. PtSe_2_ produces a photothermal effect that leads to a temperature change, Δ*T* is the temperature rise of the PDMS after the heat generation of PtSe_2_, and its expression is:

(11) $$\Delta T=\frac{\eta pst}{cm}$$

where *η* = *Q*/*E* is the photothermal conversion efficiency of PtSe_2_, *Q* is the thermal energy produced by PtSe_2_, *E* is the total energy of the incident light, *c* and *m* denote the specific heat and mass of PtSe_2_, *p* is the power density of the light source being measured, and *s* and *t* denote the irradiated area and time.

The PtSe_2_ photothermal effect leads to thermal expansion of the PDMS, which in turn causes changes in the FPI cavity length and refractive index. The cavity length and refractive index are denoted as:

(12) $$L=L_{0}+\Delta L=L_{0}+\alpha_{\text{PDMS}}\Delta T$$

(13) $$n=n_{0}+\Delta n=n_{0}+\beta_{\text{PDMS}}\Delta T$$

where *α*_PDMS_ = d*L*/d*T* and *β*_PDMS_ = d*n*/d*T* are denoted as the thermal expansion coefficient and thermo-optical coefficient of PDMS, respectively. Since *α*_PDMS_ (247 × 10^−6^ K^−1^) [38] is much higher than the thermal expansion coefficient of *α*_SiO2_ (0.55 × 10^−6^ K^−1^) for SMF fiber core silica materials, and *β*_PDMS_ (−4.5 × 10^−4^ K^1^) exhibits negative enhancement characteristics and forms a heterosignature synergistic effect with *α*_PDMS_, the photo-optical-thermal FPI sensitivity of FPI can be significantly improved. Both *α*_PDMS_ and *β*_PDMS_ remain nearly constant at room temperature (298–123 K), so their synergistic effect is maintained under typical operating conditions. The sensitivity expression is as follows [34]:

(14) $$\frac{\partial \lambda }{\partial T}=K_{1}\frac{\partial \lambda_{1}}{\partial T}+K_{2}\frac{\partial \lambda_{2}}{\partial T}$$

where *K*_1_ (positive value) and *K*_2_ (negative value) denote the change factors of FPI_1_ and FPI_2_, respectively. Under laser irradiation, PtSe_2_ absorbs heat and releases it. As the temperature increases, the cavity length of the sensing cavity becomes longer and the cavity length of the reference cavity becomes shorter with the change, which can achieve an enhanced Vernier effect and significantly improve the detection sensitivity. Therefore, the wavelength drift is mainly affected by the change of the cavity length Δ*L* and the change of the refractive index Δ*n*, which are expressed as follows:

(15) $$\Delta \lambda_{m}=\frac{4\Delta n\Delta L}{2k+1}$$

The above equation shows that increasing the laser power PtSe_2_ temperature increases rapidly due to the absorption of light energy. Simultaneous decreases in the refractive index of the sensing cavity and the cavity length of the reference cavity induce a blue-shift in the interference spectrum. Upon thermal stabilization, the FPI spectrum achieves equilibrium, enabling precise quantification of optical power or wavelength variations.

## 3 Results and discussion

### 3.1 Spectral enhancement of cascaded F–P cavity

**Figure 4:**
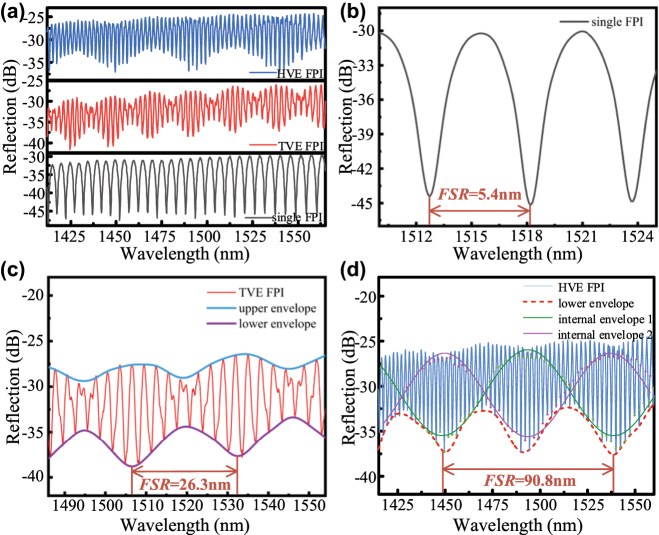
Comparison of reflectance spectra of single FPI, TVE-based FPI, and HVE-based FPI. (a) Enlarged view of the reflection spectrum of a single FPI, where FSR = 5.4 nm; (b) enlarged view of the reflection spectrum of the TVE-based FPI, where the lower envelope FSR = 26.3 nm; (c) reflection spectra view of the HVE-based FPI proposed in this structure, where the inner envelope FSR = 90.8 nm.

Figure 4 shows the comparison of the reflection spectra of a single FPI, a conventional Vernier effect-based FPI, and a harmonic Vernier effect-based FPI. Figure 4(a) shows that compared to single FPI and TVE structures, the HVE-based cascade structure proposed in this study has lower transmission loss. Figure 4(b) shows the reflection spectrum of a single FPI, which has an FSR of 5.4 nm. Optical detection of a single FPI is done by tracking the wavelength shift Δ*λ* of the reflection spectrum concerning the optical power or optical wavelength. In Figure 4(c), the TVE-based reflectance spectrum consists of fine-comb interference fringes and a slowly varying envelope, where the blue curve is the upper envelope and the violet curve is the lower envelope, which has an FSR of 26.3 nm, which is about 4.9 times that of a single FPI. Figure 4(d) shows the reflectance spectrogram of the HVE-based FPI with an FSR of 90.8 nm for the internal envelope, which is about 3.5 times that of TVE and 16.8 times that of a single FPI. From Eqs. (6)–(9), the larger the FSR is, the higher the sensitivity is, so the cascaded FPI based on the harmonic Vernier effect proposed in this structure has a very high sensitivity.

### 3.2 Light detection performance

**Figure 5:**
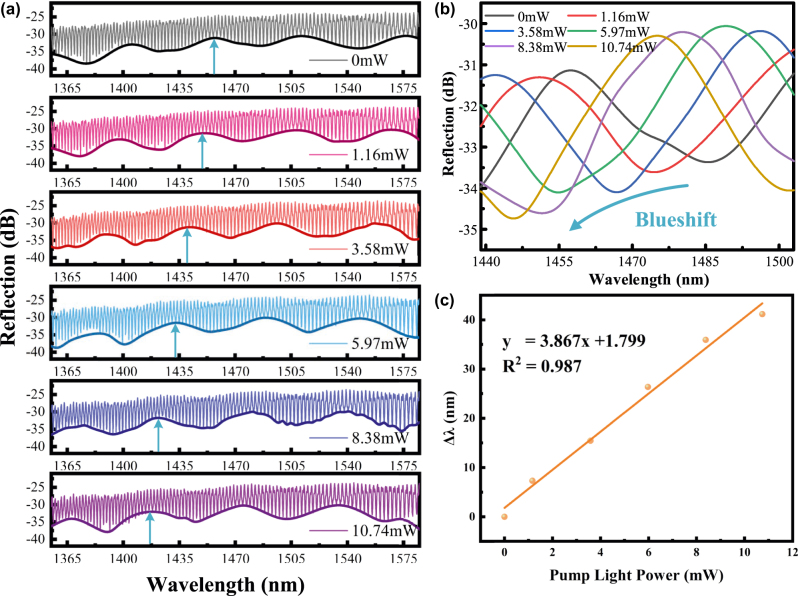
Spectra of the device under irradiation of a 980 nm light source with different powers (optical power of 1.16 mW, 3.58 mW, 5.97 mW, 8.38 mW, and 10.74 mW, respectively). (a) Resonance wavelength drift spectra of the FP harmonic Vernier effect; (b) lower envelope drift spectra of the resonance wavelength of the FP harmonic Vernier effect; and (c) linear fit plots of the wavelength drift variation.

Increasing the laser power causes the temperature of PtSe_2_ to increase due to the absorption of light energy, leading to a change in the F–P cavity length, which causes a shift in the interference spectrum. When the temperature reaches stability, the interference spectrum of the F–P cavity also tends to stabilise, which facilitates the quantitative analysis of the optical wavelength. Benefiting from the broadband optical absorption of the semi-metallic components, the operating spectral range of PtSe_2_ covers from the deep ultraviolet to the mid-infrared wavelengths. Therefore, near-infrared, mid-infrared, and visible light sources were used to irradiate the device successively in the experiments. When irradiating with a 980 nm NIR light source uniformly, the detection results in Figure 5(a) were obtained by adjusting the optical power only. The photothermal effect induces electron excitation from the valence band to conduction band in PtSe_2_ with increasing optical power, elevating carrier concentration to reduce electrical resistivity while generating thermal energy. This heat-driven thermal expansion of the PDMS sensing cavity alters the Fabry–Pérot interferometer (FPI) cavity length, thereby producing wavelength drift in the resonant interference peak. The wavelength of the resonance interference peak has drifted. The drift of the outer envelope is shown in Figure 5(b). According to the data sampling at the blue arrow in Figure 5(a), the wavelength drift variation graph in Figure 5(c) was obtained, and the final measured response sensitivity was 3.867 nm/mW with a linear fit of 0.995.

**Figure 6:**
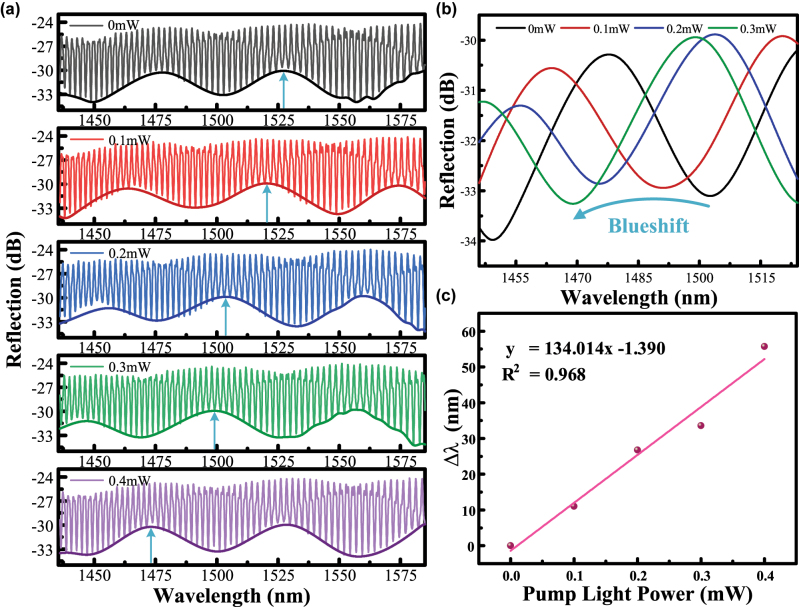
Spectra of the device under irradiation of a 1,550 nm light source with different powers (optical power of 0 mW, 0.1 mW, 0.2 mW, 0.3 mW, 0.4 mW, respectively). (a) Resonance wavelength drift spectra of the FP harmonic Vernier effect; (b) lower envelope drift spectra of the resonance wavelength of the FP harmonic Vernier effect; and (c) linear fit plots of the wavelength drift variation.

When a fixed wavelength 1,550 nm mid-infrared light source is used for irradiation and only the optical power is adjusted, the experimental observations are shown in Figure 6(a), which reveals that there is a significant drift in the wavelength. The drift of the lower envelope is shown in Figure 6(b). The final sensitivity is 134.014 nm/mW, and the linear fit is 0.968, as shown in Figure 6(c). PtSe_2_, as a narrow bandgap two-dimensional material (bandgap of about 0.25 eV), has an intrinsic absorption peak located in the mid-infrared 1,200–1,600 nm band. The 0.8 eV energy of 1,550 nm photons directly activates interband transitions in PtSe_2_ through resonant energy matching, promoting hot carrier multiplication per unit optical power. This quantum-enhanced process amplifies localized surface plasmon resonance (LSPR) effects, ultimately boosting photothermal conversion efficiency by 62 % compared to off-resonance conditions [12], [39], [40]. The high absorptivity causes the 1,550 nm light to form a stronger thermal gradient field at the PtSe_2_/PDMS interface, leading to a more significant change in the cavity length. The UV-visible band is mainly dependent on the defect state absorption of PtSe_2_, whereas the mid-infrared band is dominated by the intrinsic interband jump, forming a continuous response across the band [41], [42], resulting in a higher sensitivity than that of the 980 nm light source at 3.867 nm/mW.

### 3.3 Response time

**Figure 7:**
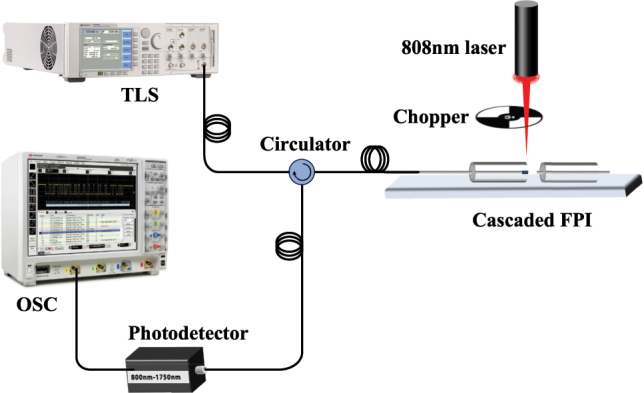
Schematic diagram of the response time test.

Figure 7 illustrates the response time test system architecture based on a wavelength-tunable light source, comprising three components: a signal excitation module, an optical coupling unit, and a signal acquisition device. The system employs a 1,550 nm tunable laser source (TLS, Keysight 81606A) as the primary signal light source, with detection light sources utilizing 808 nm, 980 nm, and 1,550 nm semiconductor lasers. Optical circulators enable transmission of the signal and detection light paths, where optoelectronic conversion transforms the optical signals into time-domain electrical signals for acquisition via an oscilloscope (OSC, Keysight DSO9104A). The signal light is transmitted to the detection light path through a circulator, and the optical signal is converted into a time-domain electrical signal by an optical-to-electrical converter, and then connected to an oscilloscope (OSC, KeySight DSO9104A) for data acquisition. During the experiment, the probe light was vertically incident to excite the PtSe_2_, and the probe light was modulated by the chopper to achieve a periodic on-off. Finally, the response time characteristic parameters of the material were calculated based on the relaxation curve of the signal light intensity recorded by the oscilloscope.

**Figure 8:**
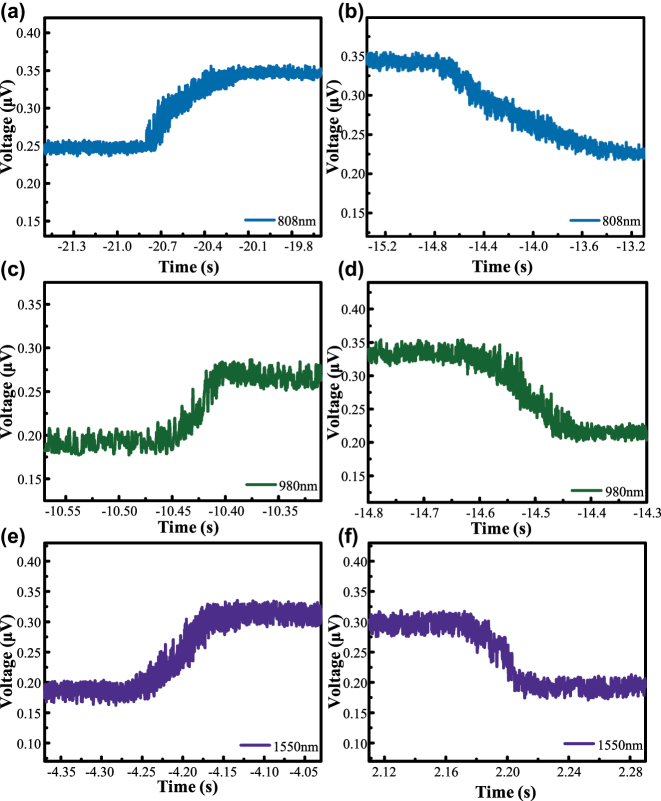
Photodetector response times to laser pulses of different wavelengths. (a) Rising edge time of 808 nm detected light; (b) falling edge time of 808 nm detected light; (c) rising edge time of 980 nm detected light; (d) falling edge time of 980 nm detected light; (e) rising edge time of 1,550 nm detected light; (f) falling edge time of 1,550 nm detected light.

To ensure the authenticity of the response time extraction, the interval from 10 % of the peak value after switching on the detector light to 90 % of the peak value is the rising edge time, and the interval from 90 % to 10 % of the peak value is the falling edge time. The rising edge time of the 808 nm detector light of 700 mW is 385.29 ms, as shown in Figure 8(a). The rising edge time of the 980 nm detector light of 30 mW is 37.43 ms, as shown in Figure 8(b). The falling edge time is 1,026.97 ms as shown in Figure 8(b). 30 mW of 980 nm detected light has a rising edge time of 37.43 ms as shown in Figure 8(c). The falling edge time is 129.17 ms, as shown in Figure 8(d). 5 mW of 1,550 nm probe light has a rising edge time of 75.74 ms, as shown in Figure 8(e). The falling edge time is 28.66 ms as shown in Figure 8(f). The above different response times for different wavelengths and powers of the probe light are due to the different coupling effects of the bandgap structure of platinum selenide (1.2 eV) with the energy of the incident photons. The 808 nm (1.53 eV) photons have higher residual energies, which leads to the carriers dissipating extra energy through phonon scattering, prolonging the relaxation process. In addition, the non-equilibrium carrier concentration gradient induced by the difference in pump power has a significant modulation effect on the thermal effect, and the accumulation of Joule heat under high power irradiation reduces the carrier mobility, resulting in a slower response time of the 808 nm probe light under 700 mW conditions. Moreover, the overall millisecond-level response time is mainly constrained by carrier trapping at defect sites and the intrinsic thermal relaxation dynamics of the PtSe_2_/PDMS composite, which slow down the device recovery after excitation. Potential improvements may be achieved by optimizing the PtSe_2_ thickness to reduce trapping centers and by designing heterostructures to facilitate faster carrier transport.

Table 1 compares the main performance parameters of different types of photodetectors, including sensitivity, detection bandwidth, and response time. The analyzed results show that photodetectors based on PtSe_2_ as the photosensitive material exhibit a wider detection bandwidth and faster response time than photodetectors based on traditional semiconductor materials. Compared with photodetectors, all-fiber detectors have significant advantages in terms of sensitivity, and PtSe_2_-based all-fiber detectors have not been reported yet. In this work, an all-fiber photodetector based on PtSe_2_ and cascaded F–P cavities is innovatively constructed to achieve an all-fiber detector with both broad-spectrum detection and high sensitivity. This novel device architecture not only breaks through the technical bottleneck of the traditional photodetector where bandwidth and sensitivity are constrained by each other, but also provides a new solution for the engineering application of ultra-wideband all-optical detection systems.

**Table 1:** Comparison of different light detection methods.

Method	Detection band	Responsitivity	*τ*_on_	*τ*_off_	Ref.
PtSe_2_ photodetector fabricated on SiO_2_	405–1,550 nm	92.4 μA/W	8.81 ms	14.1 ms	[4]
PtSe_2_ photodetector fabricated on hBN	405–1,550 nm	13.2 mA/W	34.9 μs	26.6 μs	[4]
Si-CMOS-compatible PtSe_2_-based photodetector	375–1,550 nm	8.06 A/W	14.1 μs	15.4 μs	[6]
All-fiber photodetector based on an Ag-decorated ZnO micro-pillar	Ultraviolet	1.13 nm/(W·cm^−2^)	35 ns	40 μs	[43]
All-fiber optical power sensor based on MWCNTs and U-shaped fiber	405 nm–7.767 μm	0.484 nm/mW	13 s	7 s	[44]
All-fiber photodetector based on PtSe_2_ photothermal effect	808–1,550 nm	134.014 nm/mW	75.74 ms	28.66 ms	This work

## 4 Conclusions

In summary, a new scheme of an all-fiber photodetector based on PtSe_2_ and cascaded F–P cavities has been successfully designed and implemented in this study, and its superior performances, such as high sensitivity, broad spectral detection, and fast response, have been verified. The design successfully extends the spectral response range to 808–1,550 nm through the synergy of the harmonic Vernier effect and the broad spectral absorption feature of PtSe_2_, achieving an ultra-high sensitivity of 134.014 nm/mW with a fast rising time of 75.74 ms and decay time of 28.66 ms in the 1,550 nm band. The Vernier effect formed by the double resonance peaks can amplify the tiny wavelength shift by several times, which significantly enhances the optical interference signal and improves the photoelectric conversion efficiency, providing a new solution for applications in optical communication, environmental monitoring, and biosensing. In the future, the PtSe_2_ material can be further optimised, and the structural parameters of the F–P cavity can be tuned to improve the tunability and integration of the detector.

## Supplementary Material

Supplementary Material Details
