# Supplementary material for: Highly sensitive broadband photodetector based on PtSe2 photothermal effect and fiber harmonic Vernier effect
Source: Nanophotonics. 2025 Oct 29;14(27):5023–34. doi: 10.1515/nanoph-2025-0291 (PMC12717942; doi:10.1515/nanoph-2025-0291)
Supplement: Supplementary file 1 — Supplementary Material Details [file j_nanoph-2025-0291_suppl_001.docx]

Research Article

Supplementary Material

The PtSe₂ nanosheets employed in this work (purity >99.99 wt%, Six-Carbon Technology) were characterized by Raman spectroscopy, as shown in Fig. S1(a) and (b). Three distinct peaks were observed at 170.4 cm⁻¹, 203.1 cm⁻¹, and 228.2 cm⁻¹, corresponding to the E_g_, A_1g_, and LO (longitudinal optical) vibration modes of Se atoms, respectively. The morphology and layered structure of the PtSe₂ nanosheets were further examined by transmission electron microscopy (TEM), as shown in Fig. S1(c), with the corresponding energy-dispersive X-ray spectroscopy (EDS) results displayed in Fig. S1(d). The Pt and Se peaks are clearly visible, and the atomic ratio of Pt: Se is approximately 1:2. These results confirm the high quality of the PtSe₂ nanosheets used in our experiments, which ensures reliable evaluation of their band structure–related photoresponse mechanisms.

**Fig. S1:** PtSe_2_ nanosheets: (a) Raman microscope image, (b) Raman characteristic spectrogram, (c) TEM image, (d) EDS analytical image.

The PtSe₂ nanosheets are primarily few-layer structures (~3–5 layers) with lateral sizes below 2 μm, as confirmed by TEM characterization. This assignment is further supported by Raman spectra, where the A₁g mode appears slightly stronger than the E_g_ mode, a feature commonly associated with few-layer PtSe₂ due to the enhanced out-of-plane vibrational contribution with increasing thickness [1]. They were dispersed in the PDMS colloid through ultrasonication and magnetic stirring to obtain a uniform mixture. Although some local agglomeration is visible in Fig. 2(d), the composite still exhibits stable dispersion and retains the strong intrinsic absorption of PtSe₂. These structural characteristics, together with the inherent electronic properties of PtSe₂, play a crucial role in determining the broadband response of the device. PtSe₂ exhibits a thickness-dependent band structure, undergoing a transition from a semiconducting monolayer to a semimetallic multilayer (near-zero bandgap). This tunability enables broadband absorption from visible to mid-infrared wavelengths. In our device, the PtSe₂ nanosheets provide efficient photothermal conversion due to their narrow bandgap, which aligns well with near-infrared and mid-infrared excitation. As a result, the carrier dynamics and thermal expansion of the PtSe₂/PDMS composite are strongly influenced by this band alignment, leading to enhanced sensitivity across a wide spectral range.


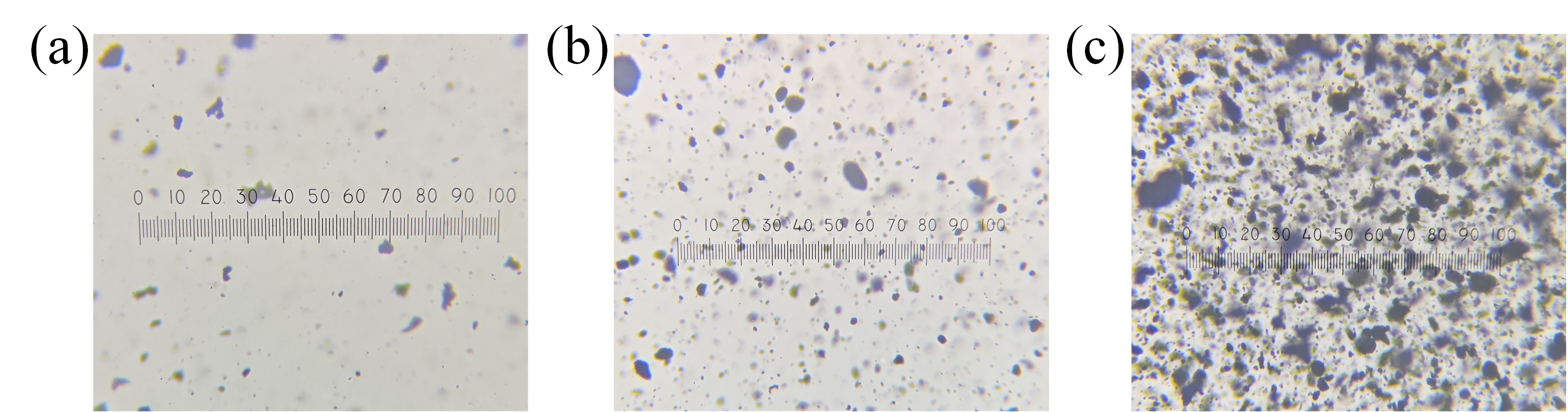


**Fig. S2:** PtSe_2_ addition concentration plots: (a) addition concentration 0.2 mg/mL, (b) addition concentration 0.5 mg/mL, (c) addition concentration 1 mg/mL.

Figure S2 shows the optical transmittance of PtSe₂–PDMS composites with three different concentrations, namely 0.2 mg/mL in Fig. S2 (a), 0.5 mg/mL in Fig. S2 (b), and 1 mg/mL in Fig. S2 (c). As the concentration increases, the composites exhibit a significant reduction in transmittance, making it difficult to obtain clear interference spectra at 0.5 mg/mL and 1 mg/mL. At the same time, higher concentrations lead to visible nanosheet agglomeration, which further affects optical uniformity. In comparison, the 0.2 mg/mL composite provides sufficient absorption while maintaining adequate transparency and good dispersion, and was therefore selected for device fabrication.

[1]C.-C. Chung, H. Yeh, P.-H. Wu, C.-C. Lin, C.-S. Li, T.-T. Yeh, Y. Chou, C.-Y. Wei, C.-Y. Wen, Y.-C. Chou, C.-W. Luo, C.-I. Wu, M.-Y. Li, L.-J. Li, W.-H. Chang, and C.-W. Chen, “Atomic-layer controlled interfacial band engineering at two-dimensional layered PtSe₂/Si heterojunctions for efficient photoelectrochemical hydrogen production,” ACS Nano, vol. 15, pp. 4627–4635, 2021.
